# The natural course of health-related quality of life in patients with clinically suspect arthralgia: a longitudinal study in progressors and non-progressors to rheumatoid arthritis

**DOI:** 10.1007/s00296-025-05865-9

**Published:** 2025-04-19

**Authors:** Sterre Bour, Lucas Goossens, Sarah Khidir, Pascal de Jong, Annette van der Helm-van Mil, Maureen Rutten-van Mölken, Elise van Mulligen

**Affiliations:** 1 Erasmus School of Health Policy & Management, Erasmus Universiteit Rotterdam, Rotterdam, The Netherlands; 2 Department of Rheumatology, Leiden University Medical Centre, Leiden, Netherlands; 3 Department of Rheumatology, Erasmus Medical Centre, Rotterdam, Netherlands; 4 Institute for Medical Technology Assessment, Erasmus University Rotterdam, Rotterdam, The Netherlands

**Keywords:** Clinically suspect arthralgia, Health-related quality of life, EQ-5D, Rheumatoid arthritis

## Abstract

Individuals with clinically suspect arthralgia (CSA) are at risk of developing rheumatoid arthritis (RA). It is unknown whether CSA patients have a deprived health-related quality-of-life (HRQoL), how this develops over time, and whether it differs between those who develop RA and those who do not. Using three unique cohorts, we explored the course of HRQoL in CSA patients and after inflammatory arthritis (IA, in this case defined as being either undifferentiated arthritis (UA) or RA) diagnosis. Longitudinal cohort data were used from two CSA cohorts (*n* = 507) and an IA cohort (*n* = 282). HRQoL as measured with the EuroQol-5 Dimensions-5 levels (EQ-5D-5 L) questionnaire was compared between CSA patients who develop IA and who do not. We estimated the course of EQ-5D-5 L scores with linear mixed models. In addition, autoantibody-positive and -negative patients were compared. As patients developed IA, their mean EQ-5D-5 L scores deteriorated with 0.12 points (0.03–0.22) over two years, with mobility and usual activities most impacted. Self-care and pain/discomfort were already impacted longer before the diagnosis. Treatment initiation after IA diagnosis showcased an improvement of 0.13 (0.09–0.16) points within 4 months, particularly in the self-care and pain/discomfort dimensions, stabilizing thereafter. In CSA patients who did not develop IA, HRQoL remained stable with a minor improvement of 0.05 (0.02–0.08) points over 2 years. Autoantibody-status had no impact. HRQoL deteriorates before IA diagnosis but improves after treatment initiation. CSA patients who do not develop IA experience stable HRQoL. These findings suggest that early intervention in CSA could prevent deterioration in HRQoL, supporting the potential value of treatment in the CSA phase.

**Supplementary Information:**

The online version contains supplementary material available at 10.1007/s00296-025-05865-9.

## Conference Abstract

Preliminary results from this study were presented as a poster with abstract at the ISPOR Europe 2023 conference held across November 2023 in Copenhagen. However, most content changed. The reference for this abstract is as follows: PCR64 EQ-5D Utility Scores in Patients With Clinically Suspect Arthralgia (CSA): Differences Between Progressors and Non-Progressors Bour, S.S. et al. Value in Health, Volume 26, Issue 12, S461.

**Supplementary Information:**

The online version contains supplementary material available at 10.1007/s00296-025-05865-9.

## Introduction

Rheumatoid arthritis (RA) is a common chronic inflammatory joint disease characterized by pain, loss of functioning, productivity losses, and increased morbidity if left untreated [1]. Measurements of generic health-related quality of life (HRQoL) in patients with RA are generally lower compared to the general population, especially on the physical health domains [2–5].

Early and aggressive treatment with disease modifying anti-rheumatic drugs (DMARDs) can mitigate disease progression and improve long-term outcomes [6]. For this reason, early diagnosis and subsequent treatment have become the cornerstones of RA management [7, 8]. In addition, the start of DMARD treatment has already been recommended for patients with undifferentiated arthritis (UA) rather than RA [9], and treatment options are even being investigated for individuals with clinically suspect arthralgia (CSA), characterized by a pattern of symptoms, including for example, pain in small joints or morning stiffness, which is found to be suspicious for developing RA by the rheumatologists [10]. CSA patients can have symptoms and limitations that are as serious as in RA patients [11–15]. Also, they may experience similar illness-perceptions as RA patients [16].

Both pharmacological and non-pharmacological strategies, such as life-style changes, could lower disease burden, and potentially delay or even prevent the onset of RA [17]. Recent pharmacological trials have demonstrated disease modification, symptom improvement, and potential prevention of RA due to treatment in the CSA phase [13, 14, 18, 19]. However, the pattern and trajectory of HRQoL in patients with CSA remain unclear, as is the impact of interventions on HRQoL in this phase. To evaluate the potential value and impact of new early-intervention strategies, it is essential to understand how HRQoL develops during in the CSA phase towards the diagnosis. Recently, two studies measured the HRQoL in CSA patients [5, 19]. However, one of the studies measured it at one time-point [5], and the other was a trial-based cost-utility analysis in which only CSA patients with subclinical inflammation that was visible on the MRI were included [19]. Longitudinal HRQoL in the whole CSA population has never been investigated, neither collectively nor separately for patients who develop clinical arthritis and those who do not. This information is essential for future cost-effectiveness models, and for guiding policymakers and treatment developers. Moreover, real-world data on changes in HRQoL can complement clinical trial data by allowing for longer follow-up and better reflecting routine practice [20]. 

Therefore, the current study aimed to investigate the natural course of HRQoL in individuals with CSA over time, as measured by the EuroQoL- 5 dimensions – 5 levels (EQ-5D-5 L) dimension scores and index score. We performed separate analyses on patients who developed inflammatory arthritis (IA, in this case defined as being either UA or RA), and those who did not. We also charted the trajectory after IA diagnosis to explore the treatment effect on HRQoL after IA development.

 [5, 13, 14, 17–20].

## Methods

### Patients

We used three data sources. For the analysis of HRQoL during CSA, we employed data from the Leiden CSA cohort [21] and the multicenter Rotterdam CSA cohort [22]. The Leiden CSA cohort was approved by the Medical Ethical Committee of the Leiden University Medical Center (#P11.210, date of approval 08-02-2012). The multicenter Rotterdam CSA cohort was approved by the Medical Ethical Committee of the Erasmus Medical Center (#MEC-2017-028, date of approval 24-03-2017). These are two independent cohorts but designed similarly. Patients were included from 2012 to 2017 onwards, respectively. Eligible patients had to have arthralgia of the small joints (symptom duration < 1 year) that was considered a potential risk for progression to RA by the rheumatologist. If another explanation for the complaints was more likely than imminent RA, patients were not included. Patients were followed for two years or until they developed clinically apparent IA, confirmed with joint swelling at physical examination by the rheumatologist. Visits were scheduled at time of inclusion, after 4 months, 12 months, and 24 months, or when patients had increased symptoms. During follow-up, CSA-patients were not treated with DMARDs (also no glucocorticoids). Since patients are still being included in both cohorts at the time of this analysis, we required a minimum follow-up time of 6 months for our analyses. We divided the CSA patients into two groups, namely (1) CSA patients who developed IA within 2 years, and (2) CSA patients who did not develop IA within 2 years. Of note, patients with CSA were not treated with DMARDs and/or glucocorticoids.

Data for the analysis of HRQoL for patients with IA were obtained from the early arthritis clinic (EAC) cohort from Leiden [23, 24]. This cohort included patients with early arthritis at physical examination by the rheumatologist and a symptom duration of less than two years from 1993 onwards. For the current study, we selected patients with IA, defined as either RA (fulfilling the 1987 and/or 2010 criteria), or UA (arthritis, but not fulfilling 1987 and/or 2010 criteria). The EAC cohort was approved by the Medical ethical review committee of the Leiden University Medical Center (#P10.108 date of approval 24-02-1993). A small proportion (4%) of the patients within the EAC cohort also participated in the Leiden CSA cohort. Visits were scheduled at time of inclusion, after 4 months, and yearly thereafter. According to Dutch treatment guidelines, patients were initially treated with methotrexate (MTX), often in combination with a short course of glucocorticoids, after IA diagnosis [25]. Patients may switch treatments after three to six months if they do not have achieved their treatment goals. Subsequent treatment may include other conventional DMARDs, biologicals or biosimilar DMARDs [25].

### Main outcome variable

Quality-of-life data were available from 2017 onwards for the Leiden and Rotterdam CSA cohorts, and from 2019 onwards for the EAC cohort. Generic HRQoL was measured with the EuroQol-5 dimensions- 5 levels (EQ-5D-5 L) questionnaire at every visit. The EQ-5D-5 L measures a person’s quality of life on five different dimensions: mobility, self-care, usual activities, pain/discomfort, and anxiety/depression. For each dimension, the health state is described by one of five levels (1: no problems, 2: slight problems, 3: moderate problems, 4: severe problems, or 5: extreme problems) [26]. Each combination of these levels is associated with a summary index value, the EQ-5D-5 L index score, which in this study was based on the Dutch value set [26]. The Dutch values of the EQ-5D-5 L range from − 0.446 (indicating the worst health state) to 1 (perfect health), with the smallest possible change being 0.022 (i.e., when moving from level 2 to 3 on the mobility dimension), though most changes in the levels of the EQ-5D dimensions exceed a value of 0.05 [27]. This value is in line with the results of a recent review, which found that the level of change that patients perceive as ‘important’ varies according to their baseline EQ-5D index score [28]. We decided to use a cut-off of 0.05-point based on the Dutch value set [27] and the insights from the review [28].

### Statistical analysis

#### The course of EQ-5D index scores

For CSA patients who developed IA, we analysed their EQ-5D-5 L scores as a function of time until IA diagnosis. We used a linear mixed model for the analysis, which also addressed missing values and the repeated-measurements character of the data [29]. Relaxing the assumption of a linear relationship between EQ-5D-5 L score and number of days till IA development, we fitted a parabolic model.

1 $$\begin{array}{l}\:EQ5D\,index\,scor{e_{ij}} = {\beta _1}Days\,to\,develo{p_{ij}}\\+ {\beta _2}\,Days\,to\,develop_{ij}^2 + \:\omega {\:_i} + \:{ \in _{ij}}\end{array}$$

In which EQ5D index score_ij_ is the EQ-5D-5 L score for individual *i* at day *j*, $\:{Date\:to\:develop}_{ij}$ is the number of days till IA development for individual *i* at day *j*, $\:{Date\:to\:develop}_{ij}^{2}$ is the squared number of days at day j for individual *i*, $\:{\omega\:}_{i}$ is the random intercept accounting for variation in EQ-5D-5 L scores between individuals, and the $\:{ϵ}_{ij}$ is the usual error term. We used an exchangeable covariance structure which assumes one common variance for all random effects and one common pairwise covariance.

For the CSA patients who did not convert to IA, we analysed EQ-5D-5 L scores as a function of time using the fixed measurements at inclusion, and 4, 12 and 24 months after inclusion. The linear mixed model was described as follows [29]:

2 $$\:EQ5D\:index\:scor{e_{ij}} = \beta {\:_1}Visit\:mont{h_{ij}} + \:\omega {\:_i} + \:{ \in _{ij}}$$

In which $\:{Visit\:month}_{j}$ was the month in which individual *i* visited the rheumatologist at time $\:j$. A similar model was used for the analysis of EQ-5D-5 L scores after diagnosis.

To provide further insight into the variation in the course of HRQoL over time we estimated the percentage of patients experiencing deterioration, improvement, or no change on the EQ-5D-5 L score with an assumed cutoff of 0.05-point [27].

#### EQ-5D dimensions

To obtain a more detailed impression of patient’s quality of life, proportions of patients with different levels of problems on each EQ-5D-5 L dimension were compared for two time-periods. Specifically, for convertors to IA we compared the period longer before diagnosis (750 to 150 days prior to diagnosis) with the period closer to diagnosis (less than 150 days prior to diagnosis). For non-convertors the comparison was made between the time of inclusion and two years after inclusion. Similarly, for patients diagnosed with IA the comparison was made between the point of diagnosis and two years after IA diagnosis.

### Subgroup analyses

We stratified patients based on autoantibody-positivity (rheumatoid factor (RF) or anti-citrullinated protein antibodies (ACPA)) [30] for convertors.

## Results

### Patient characteristics

For this study we included 507 CSA patients, 369 from Leiden and 138 from Rotterdam. From this, 61 (12%) individuals with CSA were diagnosed with IA within 2 years (convertors). The mean time from CSA to IA diagnosis was 149 days (SD 164) and almost 90% of these patients developed IA within one year.

Table 1 shows the characteristics of the CSA cohorts and EAC cohort at the time of inclusion. At baseline we had EQ-5D-5 L data from 377 CSA patients; 50 convertors and 327 non-convertors, and 207 IA patients.

**Table 1:** Baseline characteristics of patients from CSA cohorts and EAC cohort

	CSA cohorts			EAC cohort
	All patients (*N* = 377)	Convertors (*N* = 50)	Non-convertors (*n* = 327)	All patients (*N* = 207)
**Baseline characteristics**				
Female (n(%))	281 (78%)	35 (70%)	264 (79%)	125 (60%)
Age (mean (SD))	45 (12)	50 (12)	44 (12)	59 (14)
Symptom duration in days (median (25-75%)))	133 (73–310)	160 (78–365)	127 (73–288)	85 (48–182)
Duration morning stiffness > 60 min (n(%))	110 (35%)	12 (28%)	97 (36%)	91 (49%)
Difficulty making a fist (n(%))	41 (12%)	5 (10%)	35 (12%)	60 (29%)
TJC44 (median (25-75%)))	3 (1–7)	3 (0–5)	3 (1–7)	6 (3–10)
SJC44 (median (25-75%)))	-	3 (2–6)	-	4 (2–8)
DAS44 (mean (SD))	-	-	-	2.6 (1.0)
**Biomarkers (n(%))**				
ACPA+	58 (16%)	31 (62%)	27 (9%)	56 (28%)
RF+	76 (21%)	30 (60%)	46 (15%)	72 (36%)
Elevated CRP	71 (21%)	14 (31%)	56 (19%)	122 (59%)
Elevated ESR	54 (17%)	12 (28%)	41 (15%)	93 (45%)
**Disease specific outcome measures (mean (SD))**				
VAS Pain (0 = no to 100 = major problems)	40 (24)	41 (23)	40 (24)	51 (24)
VAS QoL (0 = the worst to 100 = best health state)	67 (20)	67 (18)	67 (20)	60 (22)
VAS Fatigue (0 = no to 100 = major problems)	46 (29)	42 (31)	47 (28)	48 (29)
Functional disability (HAQ-DI)	0.6 (0.5)	0.7 (0.6)	0.6 (0.5)	0.8 (0.6)
EQ-5D index score	0.69 (0.2)	0.69 (0.2)	0.70 (0.2)	0.61 (0.2)

*ACPA = anti-citrullinated protein antibodies, DAS = disease activity score, CRP = C reactive protein, CSA = clinically suspect arthralgia, EAC = early arthritis clinic, ESR = erythrocyte sedimentation rate, HAQ-DI = health assessment questionnaire disability index, QoL = quality of life, RF = rheumatoid factor, RA = rheumatoid arthritis, SD = standard deviation, SJC = swollen joint count, TJC = tender joint count, UA = undifferentiated arthritis, and VAS = visual analogue scale

In the CSA cohort, convertors and non-convertors had a slightly different mean age, with convertors being slightly older; they also had a slightly longer symptom duration before visiting the rheumatologist. However, in both groups a similar percentage of patients had morning stiffness and difficulty making a fist, and they scored similar on visual analogue scale (VAS)-pain, VAS-fatigue and HRQoL which indicated that at baseline these groups had similar complaints, (Table 1). Patients with the diagnosis IA in the EAC cohort, were on average nine years older compared to convertors from the CSA cohort. In the EAC cohort, patients generally had more complaints at baseline compared to patients with CSA.

### The course of EQ-5D index scores

**Fig. 1:**
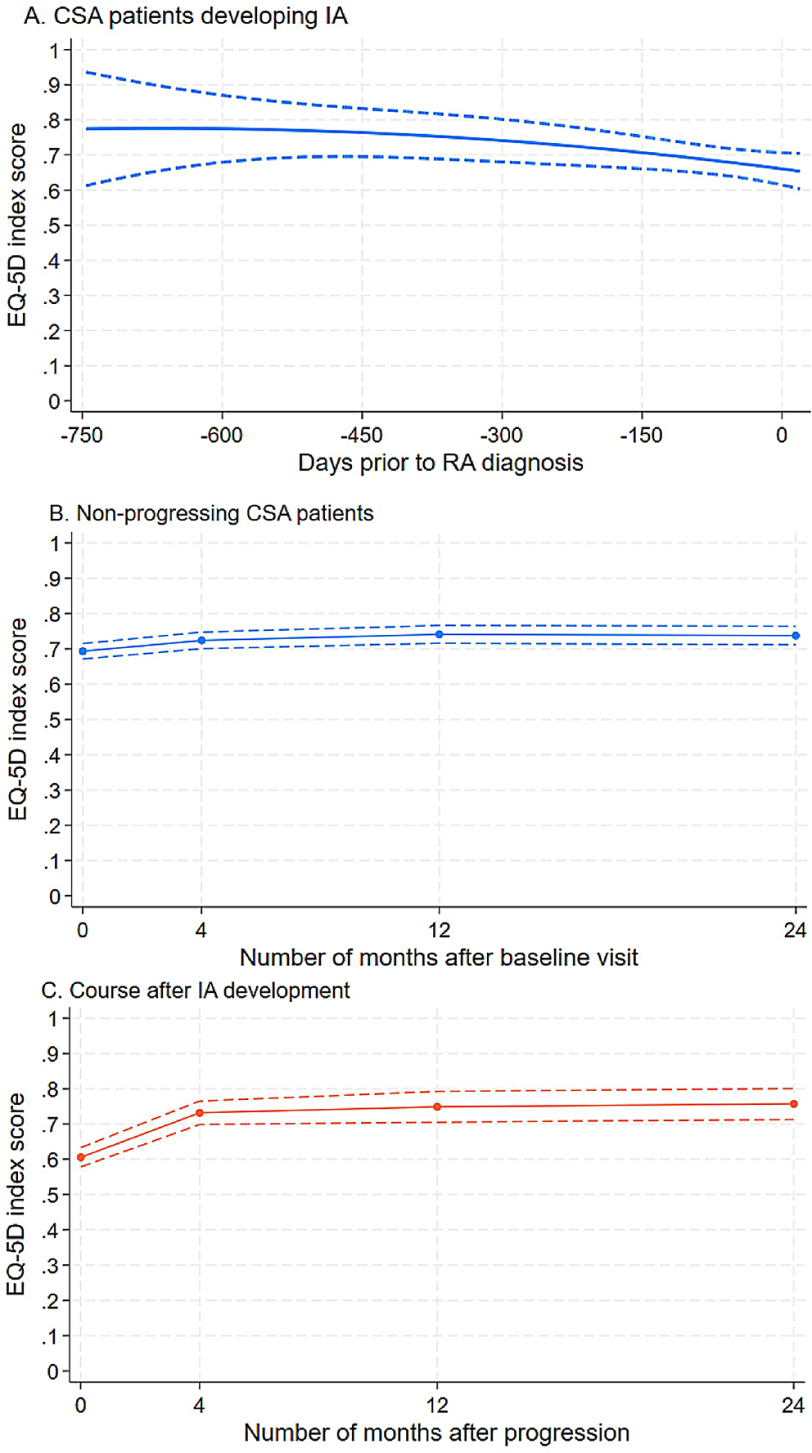
Course of EQ-5D index scores

Figure 1a shows how the mean EQ-5D-5 L score deteriorated as patients converted to IA. At the time of IA diagnosis, the mean EQ-5D-5 L score was 0.12 (95%CI: 0.03 to 0.22) points lower compared to 2 years prior to diagnosis. The deterioration occurred mostly in the last year before progression to IA.

Figure 1b shows that the course of EQ-5D-5 L scores of non-convertors was relatively stable over time. The mean EQ-5D-5 L score of non-convertors improved by approximately 0.05 (0.02–0.08) points over a course of two years. Both CSA cohorts (Leiden and Rotterdam), showed a similar trend for both convertors and non-convertors, see supplemental figures S1 and S2.

After diagnosis of IA and treatment initiation, EQ-5D-5 L scores already improved within the first 4 months after treatment initiation (Fig. 1c). The mean EQ-5D-5 L score showed an improvement of 0.15 (95% CI 0.10–0.19) points two years after IA development. The EQ-5D-5 L scores stabilized after 12 months.

Despite the clear trends, heterogeneity across patients was considerable. From the 61 patients who developed IA, we were able to assess individual courses for 28 patients from whom we had at least two measurements and a measurement close to the date of IA development. At a cutoff of 0.05 point on the EQ-5D scale, 10 patients experienced a deterioration, 7 patients improved, and the quality of life remained stable in 11 others. Similarly, from the patients who did not develop IA, 38% of patients experienced an improvement of at least 0.05 point, there was no change for 35% of patients, while 27% deteriorated, 12 months after inclusion in the CSA cohort.

However, after diagnosis of IA and treatment initiation less heterogeneity was observed; 72% of patients improved, 23% had no change, and 5% deteriorated 12 months after inclusion at a cutoff of 0.05 point.

The output from the linear mixed models can be found in supplementary tables S1 to S3.

### EQ-5D-5 L dimensions

**Fig. 2:**
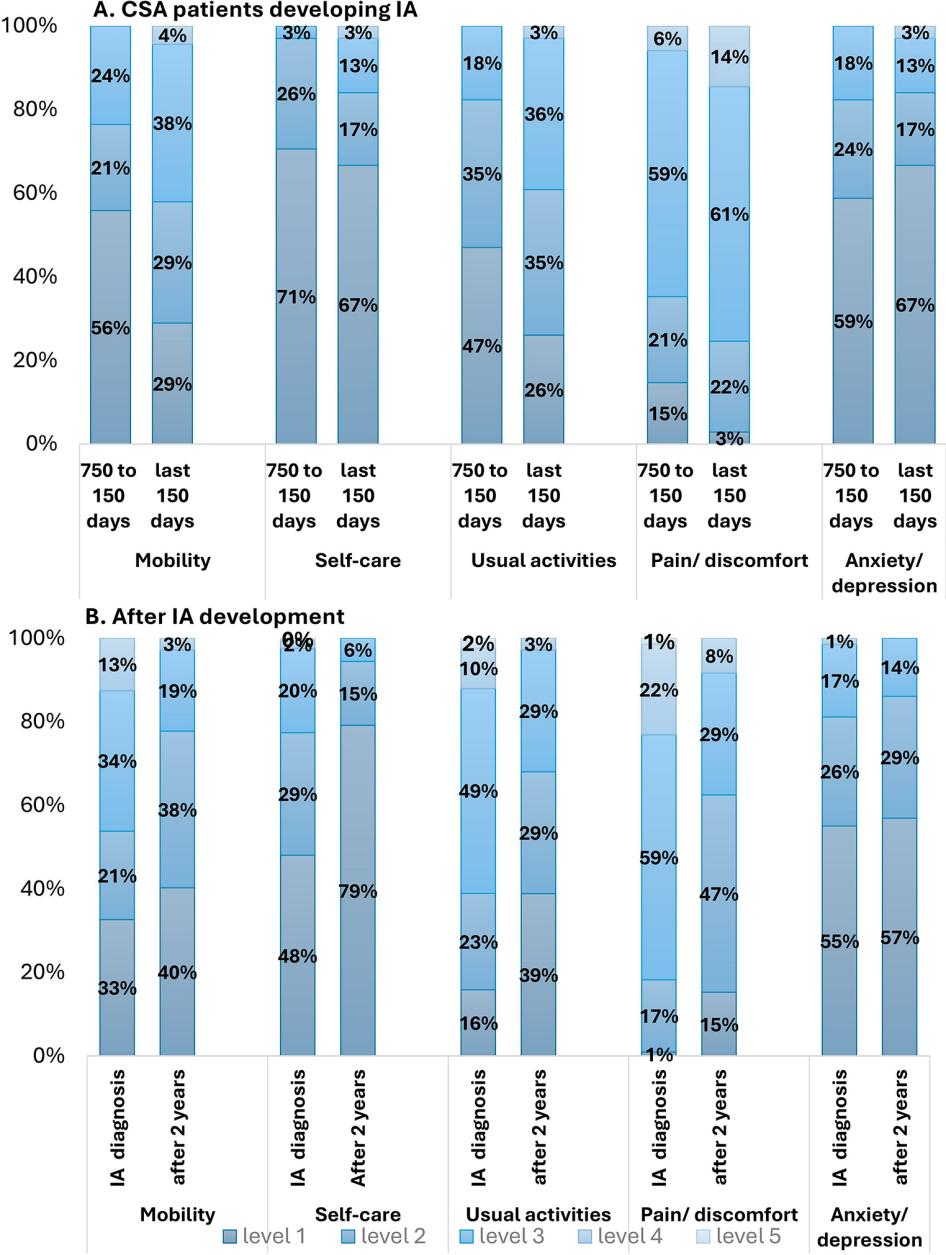
Differences in dimensions scores

The proportions of patients reporting each level of problems, on each of the EQ-5D-5 L dimensions are indicated in Fig. 2. For converters, we compared the level of problems in the period of 750 to 150 days before IA diagnosis to the period of less than 150 days before diagnosis. For patients with IA, we compared the level of problems at time of diagnosis with two years after diagnosis.

In the last 150 days prior to IA diagnosis, a higher percentage of patients indicated to have any complaints, and the complaints were more severe, especially on the mobility and usual activities dimensions (see Fig. 2a). The only dimension that did not seem to be affected by the progression to IA was the anxiety/depression dimension.

At IA diagnosis, patients had a slightly higher level of problems compared to CSA patients in the last 150 days prior to diagnosis. Especially on the self-care and usual activities dimensions, a higher percentage of patients had at least any problems, and the problems were more severe.

After diagnosis, the self-care dimension improved the most, considering that 31% more patients experienced no problems anymore two years after IA development, see Fig. 2b. Also, the pain/discomfort dimension showed a major improvement, a much higher percentage of patients had no or slight pain/discomfort (62% vs. 18%). Similar to CSA patients, the anxiety/depression dimension did not change over time.

For non-convertors, a slightly higher percentage of patients indicated to have no problems (level 1) after two years, on all dimensions. However, after two years 85% of the non-converters still had at least any problems related to pain/discomfort, 53% with usual activities, and 45% with mobility, see supplemental figure S3.

### Stratification of patients

The EQ-5D-5 L scores did not differ between autoantibody-positive and autoantibody-negative patients during the CSA phase and the early post-diagnosis phase, see supplemental figures S4 and S5. However, within the last 75 days before diagnosis autoantibody-negative patients had slightly worse EQ-5D-5 L scores compared to autoantibody-positive patients, see supplemental figure S4. After the development of IA both groups (ACPA/RF + and ACPA/RF-) were similar in terms of mean EQ-5D index scores; only at four months after diagnosis autoantibody-negative IA patients had a higher mean EQ-5D-5 L score.

## Discussion

This study investigated the natural course of generic HRQoL over time in patients with CSA. We showed that when patients develop IA, their mean EQ-5D-5 L scores deteriorated considerably over two years. Especially, the mobility and usual activities dimensions of the EQ-5D were affected by the onset of IA, while the self-care and pain/discomfort dimensions had already been impacted longer before the diagnosis. The treatment initiated after IA-diagnosis led to noticeable improvements in the EQ-5D index scores within 4 months, particularly in the self-care and pain/discomfort dimensions. These results suggest that treatment initiation with a similar treatment strategy in the CSA phase could be beneficial in preventing loss of HRQoL.

For those who did not develop IA, EQ-5D-5 L scores remained relatively stable over time with only a minor improvement over the course of 2 years. Despite differences in the course of EQ-5D-5 L scores between convertors and non-convertors, their overall mean scores during the complete follow-up were approximately similar, suggesting that they experience comparable problems, and that non-progressing CSA patients could potentially benefit from interventions at this stage as well [13, 31, 32]. However, due to heterogeneity within the CSA patient groups, additional biomarkers, patient characteristics, and subclinical inflammation markers could help identify who could potentially benefit most from treatment.

To our knowledge, no other studies have been conducted that investigate the natural course of HRQoL in patients with CSA. Progress towards diagnosis was studied with regards to subjective quality of life, fatigue and productivity losses. In line with our results, these studies found worsening outcomes as patients approached IA development [33, 34]. In addition, CSA patients in the Birmingham early arthritis cohort (BEACON) were found to have a HRQoL of 0.65 in a one-off measurement, which was slightly lower compared to our results [5]. A cost-effectiveness study of early treatment also estimated a mean baseline HRQoL of 0.65 [19].

Other studies found that CSA patients who did not develop IA improved on several outcome measures like MRI detected inflammation, VAS pain, and VAS fatigue scores [35], while they also had productivity gains [34] over a course of two years, which is not completely in line with our results. We found only a minor improvement over a course of 2 years.

For patients with an IA diagnosis, we observed a response within 4 months after treatment initiation. In line with our results, other studies found a similar improvement on the EQ-5D-3 L after treatment initiation, depending on the treatment strategy [36, 37]. Although the HRQoL improved, patients with IA still had a generic HRQoL of 0.76 after 2 years, which is lower compared to the HRQoL of 0.88 of the general population [5]. The deprived HRQoL of patients with IA could be influenced by a higher prevalence of comorbidities such as sleep disorders [38], higher risk of depression [39], and higher cardiovascular risk [40].

Our results suggested no notable differences in generic HRQoL between autoantibody-positive and autoantibody-negative CSA patients. Comparable results were previously shown for other outcome measures like pain, morning stiffness, fatigue, functional disabilities, and presenteeism [41]. Although autoantibody-negative CSA patients who developed IA had slightly more complaints than autoantibody-positive CSA patients who developed IA, the course was similar for both groups [41]. In line with our results, autoantibody-positive and autoantibody-negative patients with IA had similar scores on different outcome measures after 2 years due to the treat-to-target approach [42–44].

This study has some limitations. Firstly, the number of patients who developed IA of whom we had multiple observations and observations close to the date of IA development was limited, leading to uncertainty in estimates. However, comparing the dimension scores longer before (750 − 150 days) diagnosis (*n* = 34) and less than 150 days before diagnosis (*n* = 69) in the same patients, we clearly observe lower EQ-5D-5 L scores and more (severe) problems on the different dimensions of the EQ-5D-5 L closer to the date of diagnosis.

Secondly, a substantial proportion of non-progressing CSA patients did not complete the full two-year study period (yet), which could introduce bias into the findings. Potentially, the patients who dropped out did not drop out completely at random. If CSA patients who experienced symptom resolution decided to discontinue visits to the rheumatologist, the EQ-5D-5 L scores might underestimate actual improvement. Additionally, the CSA patients who did not complete the full two-year study period yet, could potentially still develop IA, although we showed that most patients developed IA within 1 year (~ 90%).

Thirdly, only a small proportion (4%) of patients are present both in the CSA cohorts as well as the EAC cohort. Besides, a nine-year age-gap is observed between patients who develop IA from the CSA cohorts and patients in the EAC cohort, suggesting these patients would have different characteristics or a different onset of disease [45]. The severity of inflammation is higher in the EAC with a median swollen joint count of 4 (range 2–8)), compared to a median swollen joint count of 3 (range 2–6) at the moment of IA development in the CSA cohort. Also, in terms of HRQoL the mean EQ-5D-5 L score (0.66) at the moment of IA development is slightly better than the mean EQ-5D-5 L score (0.61) at inclusion in the EAC cohort. There seems to be some discrepancy between patients with CSA at the moment of IA development and patients with IA in the EAC cohort.

We explored the generic HRQoL among patients with CSA to understand the problems CSA patients encounter across the different dimensions of the EQ-5D. The findings of this study could contribute to a cost-effectiveness analysis to evaluate pharmacological and non-pharmacological interventions during the CSA phase [17]. Such a cost-effectiveness analysis is used to inform policymakers on effective resource allocation and to facilitate physicians in optimizing care pathways for individuals with CSA.

Results from different proof-of-concept trials support treatment in the CSA phase to improve symptoms, physical functioning, and productivity (11–13), although the impact on preventing IA is still unclear. Results on the prevention of RA are mixed [13–15, 46]. One of the reasons is the ongoing challenge of predicting which individuals with CSA will progress to IA and when [47], making it difficult to determine the optimal choice and timing of an intervention [17], while also avoiding overtreatment [48]. As an illustration of this, one cost-utility only found a minor health gain of 0.04 QALY in two-years of treatment during the CSA phase, for patients selected based on the presence of subclinical inflammation as established by an MRI and of which approximately 20% of the individuals developed IA [19].

Patients who participated in such trials as well as rheumatologists seemed to be willing to initiate treatment during the CSA phase [32], even when the probability of RA development are as low as 20%. In that respect, 84% of non-convertors would recommend treatment to patients with similar complaints, as compared to 69% of the convertors to IA [32]. Another study showed that individuals at risk with arthralgia are more likely to undergo predictive testing, consider preventive medication (38% vs. 10%), and make lifestyle changes (83% vs. 20%) compared to asymptomatic individuals at risk [49].

In conclusion, the HRQoL of CSA patients who developed IA deteriorated towards the development of IA. For non-progressing CSA patients, their HRQoL was relatively stable, only a minor improvement was observed. Considering that the HRQoL improves 4 months after treatment initiation and then remains stable, interventions during the CSA phase could potentially avoid the loss of HRQoL experienced by some CSA patients.

## Electronic supplementary material

Below is the link to the electronic supplementary material.

Supplementary Material 1

## Declarations

### Disclaimer

No part of this manuscript, including the text and graphics, are copied or published elsewhere in whole or in part. No AI was used for writing and editing.
