# Supplementary material for: The natural course of health-related quality of life in patients with clinically suspect arthralgia: a longitudinal study in progressors and non-progressors to rheumatoid arthritis
Source: Rheumatol Int. 2025 Apr 19;45(5):112. doi: 10.1007/s00296-025-05865-9 (PMC12009239; doi:10.1007/s00296-025-05865-9)

Supplementary files

**Supplementary figure S1. Convertors: EQ-5D index scores for CSA cohort Leiden and CSA cohort Rotterdam.**

**Supplementary figure S2. Non-convertors: EQ-5D index scores for CSA cohort Leiden and CSA cohort Rotterdam.**


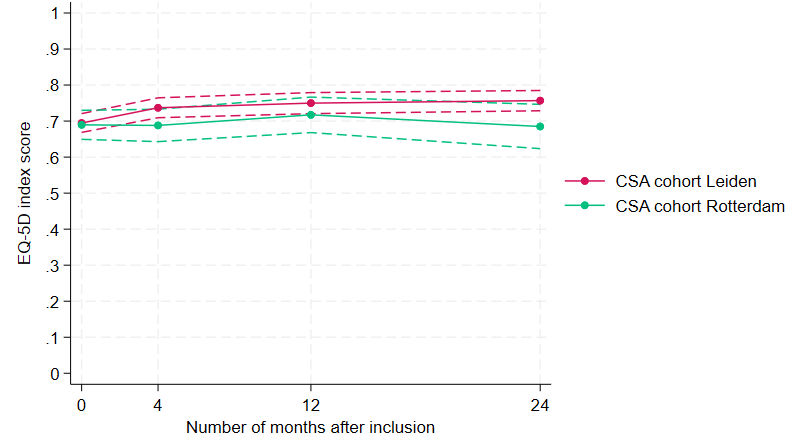


**Supplementary table S1. Linear mixed model: convertors**

|  | Beta coefficient | 95% Confidence interval | |
| --- | --- | --- | --- |
|  |  | **Lower limit** | **Upper limit** |
| Days to RA development | -0.0003 | -0.0008 | 0.0001 |
| Days to RA development² | -2.60e-07 | -9.66e-07 | 4.46e-07 |
| Constant | 0.6605 | 0.6146 | 0.7063 |

**Supplementary table S2. Linear mixed model: non-convertors**

|  | Beta coefficient | 95% Confidence interval | |
| --- | --- | --- | --- |
| Visit month |  | **Lower limit** | **Upper limit** |
| 4 months | 0.029 | 0.008 | 0.051 |
| 12 months | 0.047 | 0.023 | 0.071 |
| 24 months | 0.048 | 0.022 | 0.075 |
| constant | 0.696 | 0.674 | 0.717 |

**Supplementary table S3. Linear mixed model: After diagnosis IA, all IA patients**

|  | Beta coefficient | 95% Confidence interval | |
| --- | --- | --- | --- |
| Visit month |  | **Lower limit** | **Upper limit** |
| 4 months | **0.126** | **0.090** | **0.162** |
| 12 months | 0.143 | 0.096 | 0.190 |
| 24 months | 0.151 | 0.103 | 0.200 |
| constant | 0.606 | 0.578 | 0.633 |

**Supplementary figure S3. Changes in the dimensions of the EQ-5D for non-convertors.**


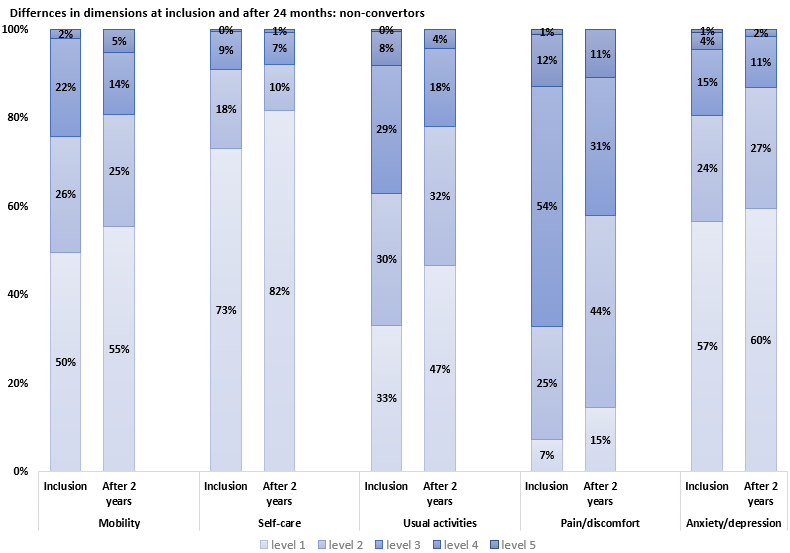


**Supplementary figure S4. Course of EQ-5D-5L index scores stratified for autoantibody-positive and -negative disease (ACPA and/or RF positivity): convertors.**

**Supplementary figure S5. Course of EQ-5D-5L index scores stratified for autoantibody-positive and -negative disease (ACPA and/or RF positivity): After IA development.**


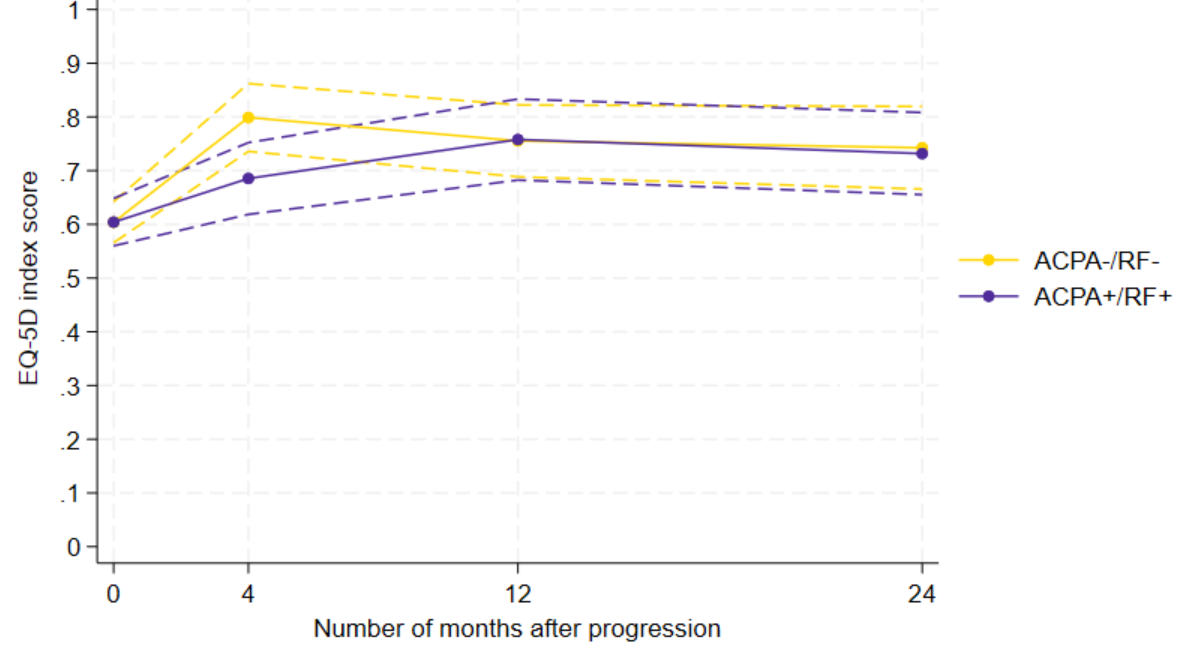

Supplement: Supplementary file 1 — Supplementary Material 1 [file 296_2025_5865_MOESM1_ESM.docx]
